# The Effects of the RANI Project on 6-Month Physical Activity Among Women Living in Rural India: A Randomized-Controlled Trial

**DOI:** 10.1089/whr.2023.0001

**Published:** 2024-06-27

**Authors:** Loretta DiPietro, Jeffrey Bingenheimer, Sameera A. Talegawkar, Erica Sedlander, Hagere Yilma, Pratima Pradhan, Rajiv N. Rimal

**Affiliations:** ^1^ Department of Exercise and Nutrition Sciences, Milken Institute School of Public Health, The George Washington University, Washington, District of Columbia, USA.; ^2^ Department of Prevention and Community Health, Milken Institute School of Public Health, The George Washington University, Washington, District of Columbia, USA.; ^3^ DCOR Consulting Pvt. Ltd, Bhubaneswar, Odisha, India.; ^4^ Department of Health, Behavior and Society, Bloomberg School of Public Health, Johns Hopkins University, Baltimore, Maryland, USA.

**Keywords:** behavioral science, community-based research, fitness, gender, intervention study

## Abstract

**Background:**

Anemia is associated with fatigue, low physical activity, and poor quality of life. The purpose of this study was to determine the effects of a field trial on 6-month change in anemia and physical activity among nonpregnant women living in rural India.

**Methods:**

The Reduction in Anemia through Normative Innovations (RANI) Project is a cluster randomized controlled trial of a social norms-based intervention to reduce anemia among women (15–49 years). Participants (*n* = 292) performed a modified Queen's College Step Test (QCST) and wore an ActivPAL accelerometer for 3 days. Hemoglobin concentrations (g/dL) were determined using a HemoCue 301 photometer. Linear regression tested the effects of the intervention on 6-month change in hemoglobin and physical activity, while adjusting for age, body mass index, education, parity, and predicted VO_2max_.

**Results:**

We observed no differences in hemoglobin (11.8 ± 1.2 vs.11.6 ± 1.4 g/dL) or overall physical activity (36.6 ± 2.1 vs. 35.3 ± 5.8 metabolic equivalent of task-hours/day) at 6 months between the treatment and control groups, respectively. In contrast, steps/day was significantly higher in the treatment, compared with the control group (β = 1353.83; 95% confidence interval: 372.46, 2335.31), independent of other covariables.

**Conclusions:**

The potential to modify walking and other health-seeking behaviors using a social norms approach is worthy of further investigation among women living in rural India.

Clinical Trial Registry – India: CTRI/2018/10/016186.

## Introduction

More than half of women of reproductive age living in India have anemia (defined as a blood hemoglobin concentration <12 g/dL).^1^ Most anemia in India can be attributed to iron deficiency (Fe <15 μg/L) because of poor dietary iron intake, low iron absorption, and iron loss due to intestinal worm infection, pregnancy, and menstruation.^2^ Anemia targets oxygen delivery to the working muscles and thus impairs aerobic capacity, while iron deficiency affects tissue oxidative capacity (*i.e*., diffusion of oxygen to the muscle mitochondria for energy production).^3^ Anemia thus is associated with fatigue, low physical activity and work productivity, and poor quality of life.^4^ This is particularly problematic because ∼55% of women living in India engage in agricultural labor, with the majority of them being of reproductive age,^5^ suggesting that most women carry a dual burden of outside labor and family care. Therefore, being physically active and physically fit has important health, financial, and social implications for these women.

For over five decades, the Indian government has promoted the use of iron–folic acid supplements (IFA) for the prevention and treatment of anemia; nonetheless, IFA use by Indian women remains low.^1^ This may be due in part to the lack of interventions focused specifically on improving the demand for and uptake of IFA and iron-rich foods.^6^

Several cultural barriers may interfere with the demand and use of IFA among Indian women. Recent qualitative research conducted in Odisha, India, indicates that women prioritize the health and wellbeing of their husbands and children over their own health.^6,7^ For example, women will eat only after everyone else in the family has eaten and thus are often left with inadequate and poor-quality food. Moreover, decisions about women's health-seeking behaviors often are imposed by their husbands or mothers-in-law, and thus they may not seek treatment for anemia.^8^ Other barriers include lack of knowledge and awareness of how prevalent anemia is in their communities (especially in nonpregnant women), as well as the types of foods that are rich in iron.

Anemia is then made worse by the fact that physical weakness appears to be part of their identity as women.^7^ Thus, changing health behavior in this Indian population requires intervention at multiple levels (individual, familial, community, and policy), thereby requiring social norms themselves to change.

The Reduction in Anemia through Normative Innovations (RANI) Project is a social norms-based intervention focused on improving IFA supplementation demand and use and is based in the theory of normative social behavior.^9^ This theory proposes that people change behaviors because they perceive that others around them are changing and they do not want to be left behind.^10,11^ There is evidence that social norms are associated with intentions to take iron supplements, as well as with actual iron supplement intake.^12,13^

Social norms also relate to other positive health behaviors. Cross-sectional evidence from adults^14–16^ and some experimental data from adolescents^17^ and older adults^18^ suggest that social norms are associated with physical activity behaviors, independent of social support.^19^ To our knowledge, however, there is no experimental evidence linking a social norms-based intervention to change in IFA use, anemia status, and consequent physical activity level. Therefore, we analyzed 6-month data from the RANI project to examine this relationship.

We hypothesized that the RANI intervention would result in greater improvements in blood hemoglobin concentrations, compared with usual care and that these improvements in hemoglobin concentrations would, in turn, be associated with increased levels of physical activity in Indian women 15–49 years of age.

## Methods

The RANI Project is a cluster randomized controlled trial of a norms-based intervention to reduce anemia among women of reproductive age in Odisha, India. The project was implemented in two blocks (Athamalik and Kishorenagar) in the Angul district of Odisha (a block is an administrative unit akin to a county in the United States). Detailed information on the cluster sampling and randomization methods are described elsewhere.^9^ Briefly, 89 clusters of villages were randomized into treatment and control groups on a 1:1 basis by the program implementers, using a random number generator. Women (2055 per group) living in 30 selected clusters (40–41 villages) were then randomly selected for data collection. All women between 15 and 49 years of age, who resided in one of the villages selected for study, and who speak Odia were eligible to participate in the larger trial. Women in the treatment arm received components of the RANI intervention, while women in the control arm received usual care.

By design, the sampling scheme also randomly generated a subset (187 per arm) of nonpregnant participants who performed the Queen's College Step-Test (QCST) to predict maximal aerobic capacity (VO_2max_) and wore a physical activity monitor for 3 days. This subset of participants was thus meant to be representative of the parent sample.

We performed the current longitudinal analysis using data from this subset of participants, all of whom had complete QCST and physical activity data at both baseline (July–September, 2019) and 6 months of follow-up (January–March, 2020; *n* = 292). Attrition from baseline appeared equal between the treatment (*n* = 20) and control (*n* = 18) groups, and was due to missing ActivPAL data at 6 months. This sample size of 292 women allowed about 80% statistical power (1-β) based on a 16% between-group difference in cognitive performance (a secondary outcome having the greatest amount of error).^9^ Data collectors, program implementers, and statisticians were blinded with regard to the treatment and control status of the villages.

Informed consent was obtained in Odia by local data collectors. In the case of participants under the age of 18 years, written permission of one parent or legal guardian and assent of the participants were obtained. All procedures were approved by Institutional Review Boards (IRB) at the George Washington University in the United States, as well as Sigma Science and Research, an independent IRB located in New Delhi, India, and the Indian Council for Medical Research's (ICMR's) Health Ministry's Screening Committee (HMSC).

### The RANI intervention

Detailed information on the components of the intervention is described elsewhere by Yilma, et al.^9^ We used a social norms approach to include factors key to behavior change at multiple levels of the socioecological model (individual, interpersonal, community, and policy). Women of reproductive age and other community members participated in group education meetings about anemia, IFA supplementation, and iron-rich foods so they could convey this knowledge to their community.

Ten learning modules were developed that included a mix of didactic learning and games focused on specific behavior changes related to IFA use, nutrition, and self-care. The intervention also included stories of overcoming barriers to IFA use through six short videos featuring members of the target audiences (women, husbands, mothers-in-law, and health workers). These videos reinforced social norms messaging that all women of reproductive age should be taking IFA throughout their reproductive life course and their family and friends should support this behavior.

As part of the intervention, point-of-care hemoglobin tests assessed anemia status of 15 women per village every month, results of which were displayed to promote community assessments of changes over time, using graphic methods appropriate for people with low literacy.

### Sociodemographic and body stature characteristics

Data on age, education (years), caste, and parity (number of children) were gathered at baseline by questionnaire. Height and weight were measured on a stadiometer and digital scale, and the body mass index (BMI: weight [kg]/height [m^2^]) was used as an indicator of body stature.

### Anemia status

Hemoglobin concentrations (g/dL) were determined at baseline and 6 months from a finger-stick using a HemoCue 301 photometer (HemoCue AB, Angelholm, Sweden). This instrument provides hemoglobin levels immediately and accurately.^20^ Anemia (yes/no) was defined as a hemoglobin concentration <12 g/dL.^21^

### Physical activity

At baseline and at 6 months, participants were asked to wear an ActivPAL (PAL Technologies, Ltd., Glasgow, UK) for three consecutive days to establish levels of daily physical activity. Prior formative research on the cultural norms of this study population indicated that 3 days was the maximal number of days that women would tolerate wearing the ActivPAL.^6^ The ActivPAL is small (53 × 35 x7 mm), light weight (15 g), and is attached to the thigh with Tegaderm, thereby making it waterproof during bathing. The ActivPAL is capable of recording continuously, and the stored activity profile is retrieved and processed afterward using a personal computer. Thus, participants were blinded from their actual physical activity data during data collection.

Data for the different behaviors are expressed as averaged hours/day of sitting, standing, reclining while awake, and reclining while sleeping, steps/day or metabolic equivalent of task (MET)-hours/day. For reference, a MET is the ratio of the metabolic (*i.e*., energy) cost of a given activity to the resting metabolic rate, and often is used as an indicator of intensity. An activity costing five METs (*e.g*., brisk walking) is performed at five times the resting metabolic rate. MET-hours/day is a summary measure of physical activity volume throughout the day, and reflects both the intensity and duration of different activities. As this is a rural, agricultural community, there was little day-to-day variation in work activity. Therefore, ActivPAL data were collected on any day of the week, weekdays, or weekend days.

### Predicted VO_2max_

To account for overall fitness level, predicted VO_2max_ was determined at baseline and 6 months using a modified QCST,^22^ which was performed on a step 12 inches in height and at a cadence of 22 steps per minute (determined using a metronome) for 3 minutes. The height of the step was reduced from 16.25 to 12 inches to accommodate the smaller stature and clothing of the participants. Heart rate (HR) was measured continuously (Polar, Finland) and was recorded while sitting before exercise, at 1, 2, 3 minutes during the test, and at 30- and 60-sec of recovery. Predicted VO_2max_ (mL∙[kg∙minutes]^−1^) was calculated as (65.81–[0.1847 × HR (bpm) measured at 30 seconds of recovery]).^23^ The QCST has demonstrated strong validity against the measurement of the Physical Fitness Index (Pfi) from the Harvard Step Test (*r* = 0.90; *p* < 0.0001)^24^ in women living in India but may slightly overestimate VO_2max_ compared with a graded exercise test using indirect calorimetry.^25,26^

### Statistical analyses

Univariate statistics (mean ± standard deviation and frequencies [%]) first were generated on all study variables. Pearson Product Moment Correlation Coefficients and independent samples *t*-tests determined the simple associations between study variables. Predetermined hierarchical regression models tested the independent effects of the RANI intervention on 6-month physical activity (MET-hours/day and daily step counts), while adjusting for baseline physical activity, change in hemoglobin concentrations, age, age,^2^ BMI, education, and parity. These covariables were chosen based on their established associations with hemoglobin concentrations and physical activity in the literature.^8,14,15,22–26^ All statistical tests were performed in STATA (v. 16.1) at an α-level of 0.05 (two-sided).

## Results

Baseline characteristics of the study population are displayed in Table 1. As indicated, women were ∼30 years of age, of small body stature, and were mildly anemic. In fact, 61% of the participants had anemia at baseline. Twenty-eight percent and 21% of women in the treatment and control groups, respectively, were members of a scheduled tribe, and the majority of them had between one and three children. Although 18% of the women in each study group had no education, between 47% and 50% had 8 or more years of schooling. At 6 months, IFA use was 27.1% and 4.1% in the treatment and control groups, respectively; yet hemoglobin concentrations did not change between baseline and 6 months in either group (treatment: 11.6 ± 1.3 to 11.8 ± 1.2 g/dL vs. control: 11.4 ± 1.3 to 11.6 ± 1.4 g/dL).

**Table 1.** Baseline Characteristics of the Study Subjects by Treatment Assignment (*n* = 292)

Variable	Treatment (***n*** = 147)	Control (***n*** = 145)
	Mean ± SD	Mean ± SD
Age (years)	30.3 ± 8.1	29.6 ± 8.4
BMI (kg/m^2^)	21.0 ± 3.3	21.0 ± 3.9
Number of children	1.6 ± 1.1	1.7 ± 1.2
Baseline hemoglobin (g/dL)	11.6 ± 1.3	11.4 ± 1.3
Education	%	%
None	18	18
1–7 years	32	35
8–13 years	50	47

BMI, body mass index; SD, standard deviation.

On average, baseline predicted VO_2max_ was high in both the treatment and control groups, indicating an excellent level of cardiorespiratory fitness in women of this age^27^ (Table 2). Overall MET-hours/day of physical activity were also high in both study groups at baseline. For reference, 36 MET-hours can be achieved by performing work activity of moderate intensity (*e.g*., 4 METs) for 9 hours over the course of the day. Time spent sitting and reclining were low, relative to more affluent and sedentary populations in the United States.^28^ Predicted VO_2max_ did not change in either group at 6 months, and neither did MET-hours/day of physical activity or time spent sitting and reclining (Table 2). In contrast, daily step counts increased significantly in the treatment, but not the control group (*p* < 0.05) and hours/day spent standing declined significantly in both study groups (*p* < 0.01).

**Table 2.** Unadjusted Physical Activity at Baseline and 6 Months by Treatment Assignment

	Treatment (***n*** = 147)		Control (***n*** = 145)	
	Baseline	6 months	Baseline	6 months
VO_2max_ (kg∙(kg∙minutes)^−1^)	42.7 ± 3.3	42.4 ± 2.9	43.1 ± 3.0	42.3 ± 2.4
MET-hours/day	36.3 ± 2.0	36.6 ± 2.1	35.9 ± 1.8	35.3 ± 5.8
Steps/day	13784.6 ± 4759.3	14970.2 ± 5267.3^*^	13057.6 ± 4358.1	13535.1 ± 4786.1
Sitting (hours/day)	6.3 ± 2.1	6.7 ± 1.7	6.7 ± 1.9	6.6 ± 1.9
Standing (hours/day)	5.7 ± 1.9	4.7 ± 1.3^**^	5.2 ± 1.8	4.6 ± 1.5^**^
Reclining (hours/day)	0.6 ± 1.0	0.6 ± 0.9	0.8 ± 1.2	0.7 ± 1.0
Sleeping (hours/day)	8.6 ± 1.8	8.8 ± 1.4	8.5 ± 1.4	8.4 ± 1.4

Data are mean ± SD reported from ActivPAL.

^*^p < 0.05; ^**^p < 0.01.

MET, metabolic equivalent of task.

Table 3 shows the independent determinants of daily step counts at 6 months. On average, women in the treatment group took over 1350 more steps/day at 6 months compared with those in the control group (*p* < 0.01) and this was not influenced by baseline BMI, predicted VO_2max_, step counts, or hemoglobin concentrations. Each milliliter∙(kg∙minutes)^−1^ unit increase in baseline VO_2max_ significantly increased step counts by 267 steps/day (*p* < 0.01), while every unit increase in BMI reduced step counts by 148 steps/day (*p* < 0.05). Hemoglobin concentrations, age, education level, and parity were not significantly associated with steps/day at 6 months.

**Table 3.** Regression Estimates Describing the Multivariable Determinants of Daily Step Counts at 6 Months (*n* = 292)

	Estimate	95% CI
Group		
Control	—	—
Treatment	**1353.83**	**372.46 to 2335.31**
Age (years)	31.90	−50.52 to 114.32
Age^2^	0.26	−7.29 to 7.71
Baseline VO_2peak_ (mL∙(kg∙minutes)^−1^)	**266.81**	**97.01 to 436.78**
BMI (kg/m^2^)	**−148.44**	**−294.56 to −2.23**
Baseline steps/day	**0.44**	**0.31 to 0.56**
Parity	236.92	−316.40 to 790.37
Education		
None	—	—
1–7 years	−88.01	−1513.89 to 1337.87
8–13 years	−1128.82	−2639.89 to 382.23
Baseline hemoglobin (g/dL)	42.25	−386.23 to 470.72
6-month change in hemoglobin	−182.32	−649.38 to 284.75

Data in bolded print are statistically significant at *p* < 0.05.

95% CI, 95% confidence interval.

## Discussion

Physical activity (steps/day) increased after 6 months of the RANI intervention in our sample of rural Indian women; however, these improvements were not mediated by changes in hemoglobin concentrations as we hypothesized. Moreover, daily step counts increased without a change in overall MET-hours/day and this increase was independent of a primary physiological driver of physical activity; namely, predicted VO_2max_.

Rimal, et al.,^29^ recently published 6-month findings from the entire RANI cohort indicating that intervention-related changes in descriptive and collective norms (but not injunctive norms) were significantly associated with improvements in self-reported iron and folic acid consumption. Similarly, Sedlander et al.^30^ reported high fidelity to the RANI treatment protocols at 6 months. The intervention increased the odds of self-reported IFA use by 17-fold (odds ratio = 16.94; *p* < 0.001) and these odds increased in a dose–response manner with increasing exposure to each component of the intervention. Unfortunately, increased IFA use had little effect on hemoglobin concentrations at 6 months.

Observational studies of both animals and humans indicate that iron deficiency and anemia impair physical performance and work capacity^3^; yet findings from randomized, controlled trials of IFA supplementation are inconsistent.^31^ Two meta-analyses examined the role of IFA in improving work capacity in women of reproductive age.^32,33^ Pasricha et al.^32^ analyzed data from 22 trials and reported significant mean differences in VO_2max_ and in submaximal exercise performance (HR required to achieve a specific workload) between treatment groups following IFA among women with and without anemia. These findings were more pronounced in women who were iron deficient and who were trained athletes; however, it was not clear if the improvements in exercise performance included enhanced hemoglobin concentrations in the causal pathway. More recently, a meta-analysis of 18 trials in iron-deficient, nonanemic adults^33^ reported that iron supplementation significantly improved hemoglobin concentrations, as well as self-reported fatigue, without improvements in objective measures of physical capacity (including VO_2max_).

In our study population, baseline predicted VO_2max_ in both the treatment and control groups indicated an excellent level of cardiorespiratory fitness based on normative data^27^ and perhaps there was little room for improvement with IFA supplementation. Moreover, a previous analysis of baseline data in these same women^34^ indicated no association between hemoglobin concentrations and predicted VO_2max_ determined by the QCST. Altogether, these findings suggest that it may take longer than 6 months for IFA use to result in measurable improvements in hemoglobin concentrations^35^ and therefore, any increases in aerobic capacity or in overall physical activity (MET-hours/day) that may be mediated by improved anemia status would not be apparent from these current data.

The fact that overall MET-hours/day did not change despite increases in daily walking may be due to subtle changes in other behaviors, such as increased sitting/reclining or decreased standing that may not be apparent when MET-hours are averaged over the 3 days. We observed a significant decline in hours/day spent standing in both study groups that was greater in the treatment (−1.0 hour/day [Δ18%]) than in the control (−0.6 hour/day [Δ12%]) group. The energy cost of standing while doing light-intensity household chores (*e.g*., cooking) is about 2.5 METs and a 1-hour drop in standing time (−2.5 MET-hours) can easily be made up with bouts of light-intensity walking, which also cost 2.5 to 3.0 METs.^36^ Furthermore, overall daily physical activity in this rural, agricultural community is already very high (about 36 MET-hours/day), which is well above the current WHO guidelines of 150–300 minutes/week of moderate-intensity activity (or about 10–20 MET-hours/week).^37^ Importantly, the demands of agricultural labor require a high work capacity (*e.g*., VO_2max_) and high levels of physical activity to ensure the work productivity necessary for survival.

A compromised work capacity due to anemia may affect both the quantity and quality of time allotted to other family responsibilities (cooking or child care) and to leisure activities that allow for recovery from agricultural work.^3,4^ Thus, it is likely that any increases in one type of physical activity in these women of reproductive age will result in a decrease in other physical activity behaviors to conserve energy.

The very nature of the RANI intervention may account for the increased walking in the treatment group, as these women walked back and forth to RANI group education meetings, video screenings, anemia testing, etc. and increased their social interaction within their village.^38^ This type of walking behavior (separate from work-related walking), combined with greater social contact can be health promoting if, indeed, it is perceived to be pleasurable and relaxing. As stated previously, inequitable gender norms may make it very difficult for women to walk about alone and socialize during the course of the day and also suggest that women tend not to prioritize their own health, relative to the health of their family.^6,7^

The RANI intervention addressed these inequities through health education videos that prominently featured women, husbands, and mothers-in-law, in efforts to overcome barriers to health-seeking behaviors—especially IFA use. Indeed, by participating in these intervention-driven activities, participants socialized with others and engaged in discussions about various topics beyond those promoted by the IFA intervention itself.^38^

In doing so, the intervention may have led to positive behavior changes among participants, with increased leisurely walking being one of them. It is not clear, however, whether these improvements in walking behavior are sustainable once the intervention ends.

There are several strengths to this study; namely, the randomized, controlled study design and a sampling scheme that produced a representative sample of women of reproductive age living in the Angul district. Also, maximal aerobic capacity and several different dimensions of physical activity were measured objectively using validated methods.

We note, however, that due to the small stature of our participants and the fact that they wore a sari while performing the QCST, the height of the step was lowered from 16.25 inches to 12 inches. This adjustment allowed all participants to complete the test; however, it may have overestimated maximal aerobic power. Since predicted VO_2max_ was similar between the two study groups and did not change in either group over 6 months, this potential overestimation would have no effect on our findings. Despite the reported 6-month increase in IFA use,^29,30^ hemoglobin concentrations did not improve. It is possible that the IFA dose was not sufficient in magnitude or duration to produce a corresponding increase in these concentrations. On the other hand, IFA use among the treatment group may have been differentially overreported because of a social desirability bias.

Baseline data collection occurred during the rainy season (July–September, 2019), but 6-month data collection (January–March, 2020) did not. Any bias due to seasonality would have affected both groups similarly, however, and therefore would not alter our findings. Also, the first cases of COVID-19 were reported in late December of 2019 and may have affected 6-month data collection between January and February of 2020. Again, however, any disruptions due to the pandemic would have affected both study groups in a similar manner. Finally, we lost 38 participants from the study between baseline and6 months. Although attrition was nondifferential between the study groups, this loss was due entirely to missing ActivPAL data. A sensitivity analysis indicated that there were no differences in baseline step counts, VO_2max_ or any of the other covariables between those who were and were not included in the present analysis (Supplementary Table S1).

The RANI social norms-based intervention resulted in increased walking behavior that was independent of improvements in hemoglobin concentrations, overall physical activity, and maximal aerobic capacity in women of reproductive age living in rural India. The potential to modify walking behavior using a social norms approach among women living in rural India is worthy of further investigation, especially as it may relate to change in other health-seeking behaviors. These findings are particularly relevant in areas where inequitable gender norms may impede health promotion efforts for women.

## Supplementary Material

Supplementary Table S1

## Abbreviations Used

95% CI: 95% confidence interval
BMI: body mass index
HMSC: Health Ministry's Screening Committee
ICMR's: Indian Council for Medical Research's
IFA: iron–folic acid supplements
IRB: Institutional Review Boards
MET: metabolic equivalent of task
Pfi: Physical Fitness Index
QCST: Queen's College Step Test
RANI: Reduction in Anemia through Normative Innovations
SD: standard deviation
