# Supplementary material for: The Effects of the RANI Project on 6-Month Physical Activity Among Women Living in Rural India: A Randomized-Controlled Trial
Source: Womens Health Rep (New Rochelle). 2024 Jun 27;5(1):522–9. doi: 10.1089/whr.2023.0001 (PMC11257113; doi:10.1089/whr.2023.0001)
Supplement: Supplementary Table S1 [file whr.2023.0001_supplemental_table_a.docx]

| **Supplemental Table A. Baseline comparison of women with and without ActivPAL data at 6-months.** | | | |
| --- | --- | --- | --- |
|  | **Retained (n=292)** | **Missing (n=38)** |  |
|  | **Mean±SD** | **Mean±SD** | **p-value** |
| Age (years) | 29.9±8.3 | 29.3±9.5 | 0.649 |
| BMI (kg/m^2^) | 21.0±3.6 | 20.8±3.3 | 0.684 |
| VO_2max_ (ml∙(kg∙min)^-1^ | 42.9±3.1 | 42.9±3.4 | 0.977 |
| Parity | 1.6±1.2 | 1.6±1.4 | 0.902 |
| Hemoglobin (g/dL) | 11.5±1.3 | 11.4±1.7 | 0.641 |
| Steps/Day | 13416.8±4477.3 | 13492.1±5319.1 | 0.924 |
|  | **%** | **%** |  |
| Education |  |  | 0.767 |
| None | 18 | 21 |  |
| 1-7 years | 34 | 37 |  |
| 8-13 years | 48 | 42 |  |
|  |  |  |  |
| Member of a scheduled tribe |  |  | 0.421 |
| Yes | 24 | 18 |  |
| No | 76 | 82 |  |
|  |  |  |  |
| Group |  |  | 0.791 |
| Treatment | 50 | 53 |  |
| Control | 50 | 47 |  |
|  |  |  |  |
| IFA use |  |  | 0.600 |
| Using | 5 | 3 |  |
| Not Using | 96 | 97 |  |
